# Two *Bombyx mori* acetylcholinesterase genes influence motor control and development in different ways

**DOI:** 10.1038/s41598-017-05360-7

**Published:** 2017-07-10

**Authors:** Xinhai Ye, Liwen Yang, David Stanley, Fei Li, Qi Fang

**Affiliations:** 1 Ministry of Agriculture Key Lab of Molecular Biology of Crop Pathogens and Insects, Zhejiang University, 866 Yuhangtang Road, Hangzhou, 310058 China; 2 Department of Entomology, College of Plant Protection, Nanjing Agricultural University, Nanjing, 210095 China; 3 Biological Control of Insects Research Laboratory, USDA/ARS, Columbia, MO 65203 USA

## Abstract

Among its other biological roles, acetylcholinesterase (AChE, EC 3.1.1.7), encoded by two *ace* in most insects, catalyses the breakdown of acetylcholine, thereby terminating synaptic transmission. *ace1* encodes the synaptic enzyme and *ace2* has other essential actions in many insect species, such as *Chilo suppressalis* and *Plutella xylostella*. The silkworm, *Bombyx mori*, has been domesticated for more than two thousand years and its *ace*s have no history of pesticide exposure. Here, we investigated the functional differences between two *ace* genes, *BmAce1* and *BmAce2*, in the silkworm. qPCR analysis indicated that *BmAce1* is highly expressed in muscle and *BmAce2* is more ubiquitously expressed among tissues and enriched in the head. Both genes were separately suppressed using chemically synthesized siRNAs. The mRNA abundance of the two *ace* genes was significantly reduced to about 13% – 75% of the control levels after siRNA injection. The AChE activities were decreased to 32% to 85% of control levels. Silencing *BmAce2* resulted in about 26% mortality, faster and higher than the 20% in the si*BmAce1*-treated group. Silencing *BmAce1* impacted motor control and development to a greater extent than silencing *BmAce2*, although both treatment groups suffered motor disability, slowed development and reduced cocoons. Both genes have essential, differing biological significance.

## Introduction

Acetylcholinesterase (AChE, EC 3.1.1.7) hydrolyzes the neurotransmitter acetylcholine into acetate and choline with a very high catalytic activity of about 25,000 molecules per second. Mammalian AChE is encoded by a single gene, *ace*, whereas some invertebrates have multiple *ace*s. The situation is otherwise in insects because two *ace*s were discovered in *Aphis gossypii* ^1^, *Schizaphis graminum* ^2^, and *Anopheles gambiae* ^3^. Since then, the presence of two *ace*s have been confirmed in many insect species, including the mosquitoes *Culex pipiens* ^4^, *Aedes aegypti* ^5^, *Culex tritaeniorhynchus* ^6^, the aphids *Myzus persicae* ^7^, *Rhopalosiphum padi* and *Sitobion avenae* ^8^, the cockroach *Blattella germanica* ^9^, the lepidopterans *Helicoverpa assulta* ^10^, *Plutella xylostella* ^11^, *Chilo suppressalis* ^12^ and *Cnaphalocrocis medinalis* ^13^, the whitefly *Bemisia tabaci* ^14^ and the red flour beetle, *Tribolium castaneum* ^15^, to name some of them. It was suggested that two *ace* genes may have originated from gene duplication before insect speciation^15, 16^. However, outside of mosquitoes, *ace2*, but not *ace*1, have been reported in the model insect *Drosophila melanogaster* and other *Drosophila* species. Further analyses indicated that the orthologs of *ace1* were lost in the Cyclorrapha, considered an unranked taxon by some authors and a dipteran suborder by others^4^.

The biological functions of the two *ace* genes have been assessed^17^. Generally, AChE1 is the major enzyme in insects, which is more abundant than AChE2. The expression of *ace1* is higher than *ace2* in some insect species^9, 10, 18, 19^, while the opposite is true in others. The AChE2 in *B. mori* and *Apis mellifera* is the major enzyme in synaptic transmission^20, 21^. Although AChE1 is regarded as the main target of organophosphorus (OP) and carbamate pesticides, resistance-associated mutations have been reported for both genes^3, 10, 11, 18, 19, 21–24^. Separate gene silencing experiments with *B. germanica* indicate that *Bgace1* encodes the predominant AChE. We infer that while both genes are necessary, *ace1* predominates in some, but certainly not all insect species.

AChE has many functions beyond acetylcholine hydrolysis^25, 26^. In mammals and zebrafish, AChE regulates cell-matrix interactions in bone^27^. In nervous tissues, it influences neuroblastoma cell adhesion and neurite outgrowth^28^. It acts in neocortical development by the alternative splicing of its own gene^29^. AChE mediates axonal growth^30^, synaptogenesis, memory formation, stress responses, cell proliferation and apoptosis^31^. Insect AChEs also function in development. Silencing larval *H. armigera aces* with dietary siRNAs led to high mortality, growth inhibition, malformation and drastically reduced fecundity, indicating to us the *ace*s are multi-functional genes^32^. Silencing the two *ace* genes led to different sensitivity to insecticides^33^. Silencing the larval *ace*s in *Chilo suppressalis* and *P. xylostella* showed that *Csace1*, *Pxace1* and *Pxace2* have non-typical functions in regulating larval growth and motor control^12, 34^. In our view, AChEs are pleiotropic genes with a wide range of biological significance.

As seen in other species, of the two *B. mori* genes, *BmAce2* is more highly expressed than *BmAce1* at the whole animal level, at different developmental stages^20^. The differing transcripts levels of these genes suggest to us they probably have divergent biological functions in the life cycle. In this paper, we posed the hypothesis that, in addition to synaptic transmission, *BmAce1* and *BmAce2* perform physiological functions in survival, larval development. Here we present and discuss the outcomes of experiments designed to test our hypothesis.

## Results

### Knockdown of *BmAce1* and *BmAce2*

**Figure 1:**
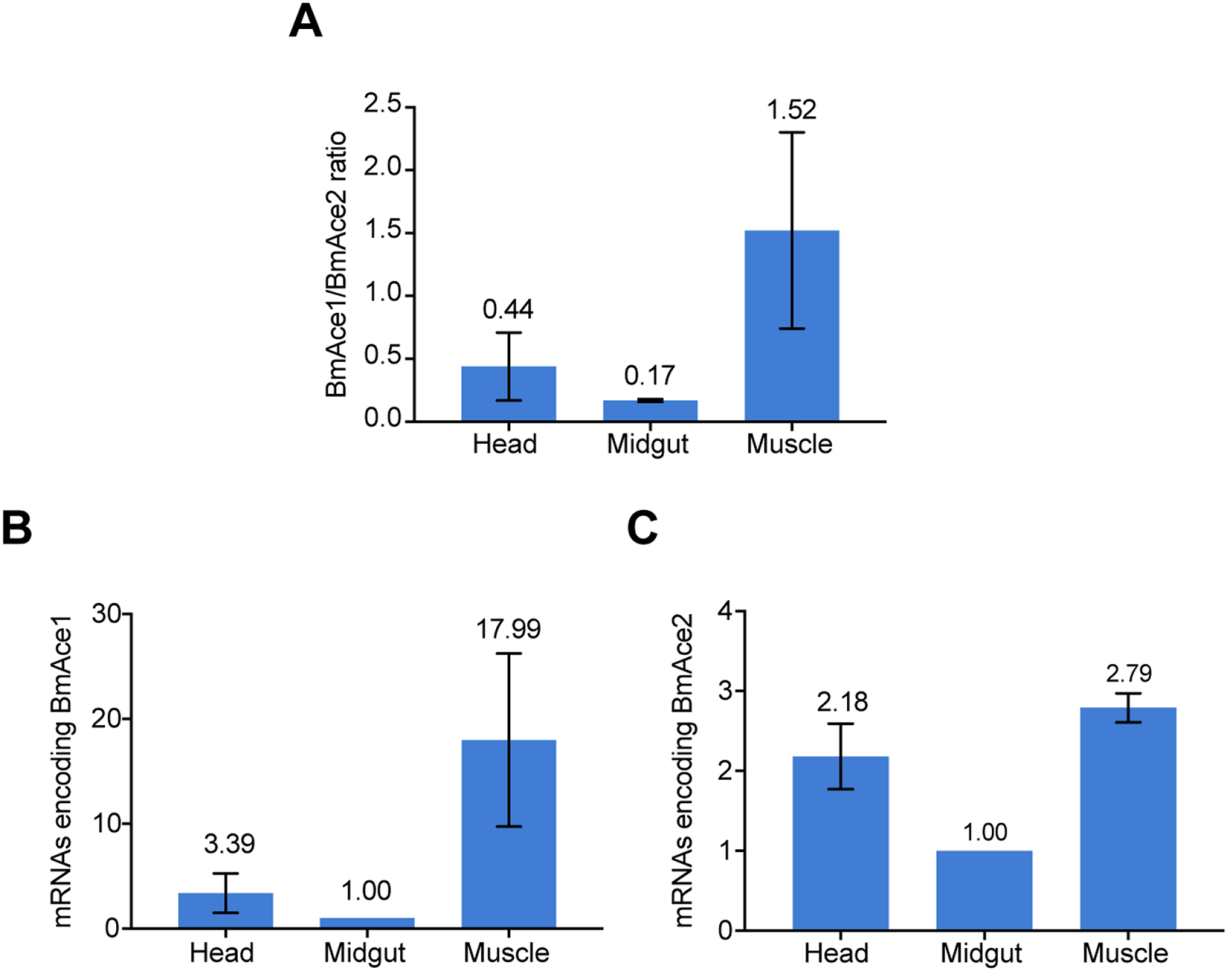
The relative abundance of mRNAs encoding two *ace*s in head, midgut and muscle. Panel A: the ratios of *BmAce1*/*BmAce2* mRNA abundances. The histogram bars represent the ratios graphically and the numbers atop the bars show the actual ratios. The error bars indicate 1 SEM. Panel B: the relative abundances of *BmAce1* in the indicated tissues. Panel C: the relative abundances of *BmAce2* in the indicated tissues. The histogram bars in Panels B and C depict the relative accumulation of mRNA encoding the indicated genes and the error bars indicate 1 SEM. The number shown above each bar represents the mean transcript levels of *BmAce1* and *BmAce2*. Ribosomal Protein 49 gene (*RP49*) was used as the reference gene for qPCR.

We designed gene-specific small interfering RNAs (siRNAs) with fluorescence-labeling to record the spread of the constructs in the silkworms under fluorescence microscopy. The injected siRNAs spread mainly to the head (Supplementary Fig. 1), where *ace*s are highly expressed. Compared to the transcript abundances in midguts, *BmAce1* transcripts accumulated to about 18-fold higher in muscle and 3.4-fold higher in heads. Similarly, the relative abundances of *BmAce2* transcripts was 2.8-fold higher in muscle and 2.1-fold higher in heads, again, compared to midguts (Fig. 1). We reported that the *BmAce2* had higher expression level than *BmAce1* during development at the whole-animal level^20^. Here, at the tissue level, *BmAce1* mRNA was more abundant than *BmAce2* in muscle (Fig. 1A).

**Figure 2:**
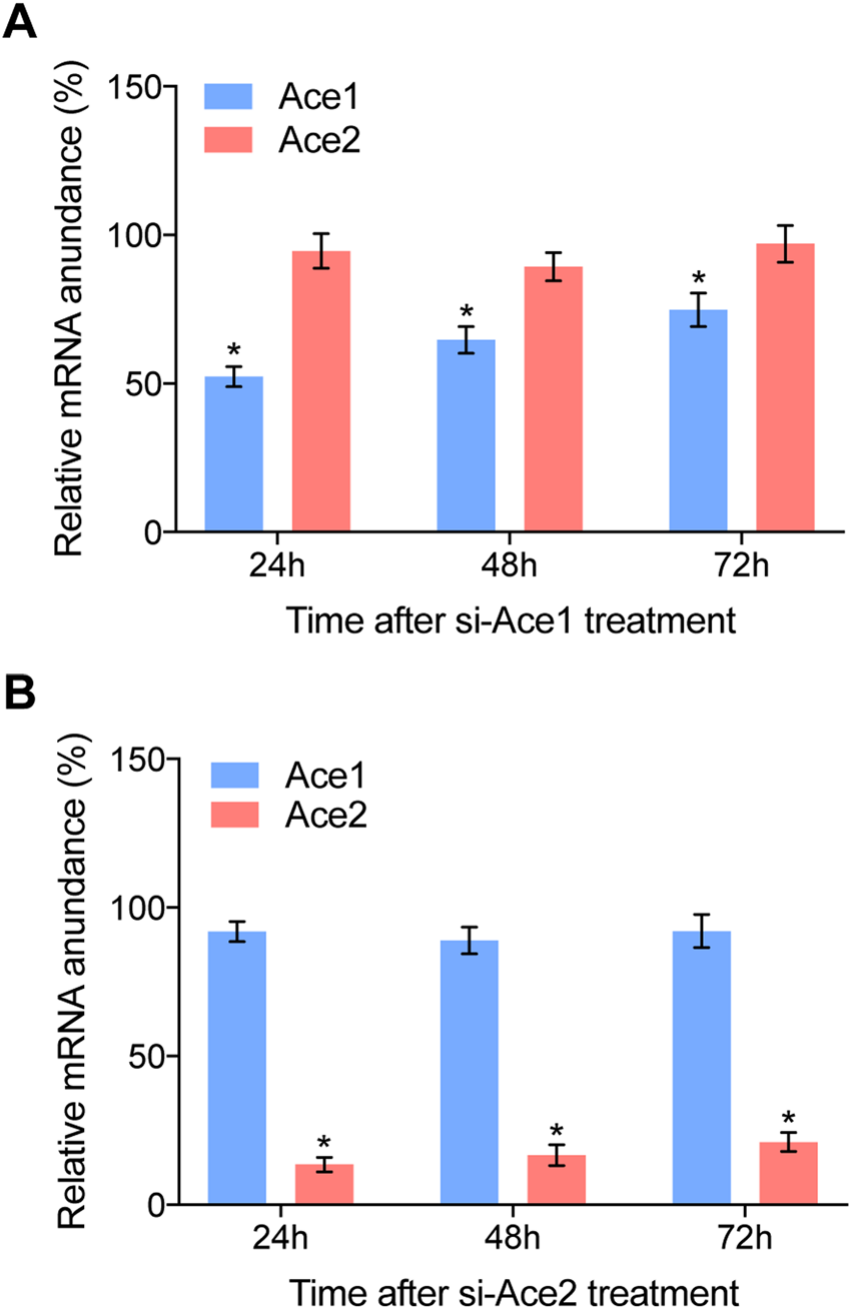
siRNA treatments led to reduced abundances of mRNA transcripts of *BmAce1* and *BmAce2* in silkworm. Panel A: siAce1 treatment led to decreased mRNAs encoding *BmAce1*, but not *BmAce2*. Panel B: siAce2 treatments led to reduced accumulation of mRNAs encoding *BmAce2*, but not *BmAce1*. For both panels, the histogram bars represent mRNA abundances and the error bars indicate 1 SEM. * Significant difference at P ≤ 0.05. All experiments were repeated in triplicate, n = 30.

To investigate the functional differences between the two *ace*s, we injected siRNAs into the day 1, third instar larvae. Negative control siRNA was a random shuffled sequence lacking sequence identities shared with si*BmAce1*. The transcript abundance of *BmAce1* decreased to about 52%, 64% and 75% of the control levels at 24 h, 48 h and 72 h post injection (PI; t-test, p < 0.05). si*BmAce2* treatments led to decreased mRNA abundances encoding *BmAce2*, by approximately 13%, 16% and 21% of the control levels at 24 h, 48 h and 72 h PI (t-test, p < 0.05). Neither siRNA construct influenced accumulation of mRNA encoding the counterpart gene (Fig. 2), showing that each *ace* was knocked down without influencing the other.

### RNAi treatments led to decreased AChE activities

**Figure 3:**
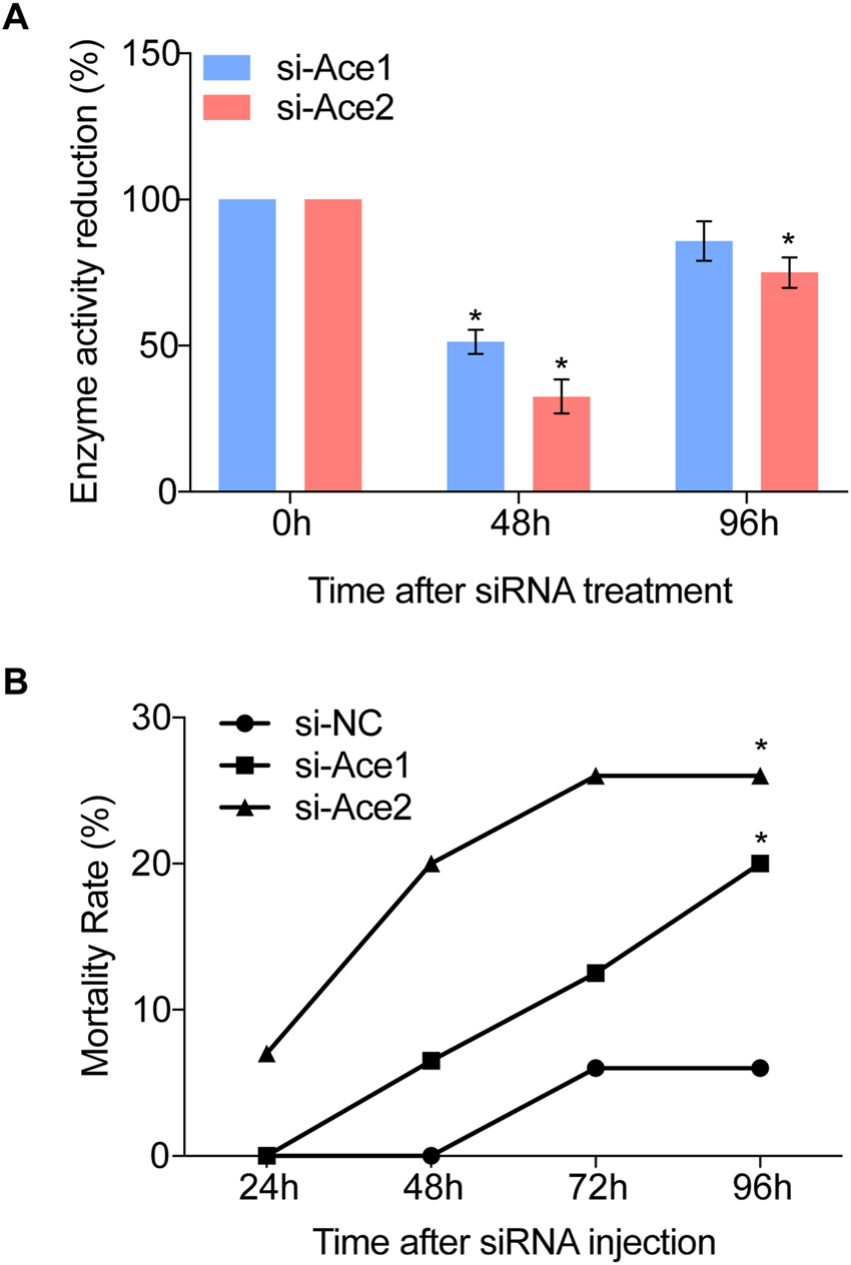
AChE activities and mortality in experimental larvae. Panel A: siRNA treatments led to reduced AChE activities at the indicated times PI. The histogram bars represent mean percent enzyme activity normalized to zero h PI. * Significant difference at P ≤ 0.05. Panel B: Mortality as a function of time PI. At all times after 24 h PI, si*BmAce1*- and si*BmAce2*-treated silkworms had higher mortalities compared to controls. Each data point indicates mean mortality and the error bars show 1 SEM. The asterisks above the bars indicate significant differences. All experiments were repeated in triplicate, n = 30.

We measured AChE activities at 48 h and 96 h PI. Compared to controls, AChE activities were reduced to about 51% at 48 h (t-test, p < 0.05) and 85% at 96 h PI in the si-*BmAce1* treatment group. Similarly, enzyme activities were reduced to about 32% at 48 h (t-test, p < 0.05) and 75% at 96 h PI (t-test, p < 0.05) (Fig. 3A). The RNAi efficiencies were different for two *ace* genes but the AChE activities were similar. We reasoned that this relates to the point that the enzyme activities are determined by the protein contents, not the mRNA levels. Among several possibilities, enzyme degradation rates may differ between the proteins and/or post-translational modifications may lead to different activity levels. The data support our view that the siRNA treatments led to reduced AChE activities, particularly at 48 h PI, despite the differences in RNAi efficiencies.

### Mortality in siRNA-treated silkworms

Silencing the two genes led to tremors, paralysis and death in some of the tested silkworms. At 24 h to 96 h PI, mortalities increased from 0 to 20% in the si*BmAce1* treatment group, and from 7% to 26% in the si*BmAce2* group (Fig. 3B). Because *BmAce2* was more highly expressed in the head than *BmAce1*, knockdown of *BmAce2* resulted in higher mortalities. It remains possible that the higher mortalities induced by si*BmAce2* is related to its better RNAi efficiency.

### *Ace* gene knockdown impaired motor ability

**Figure 4:**
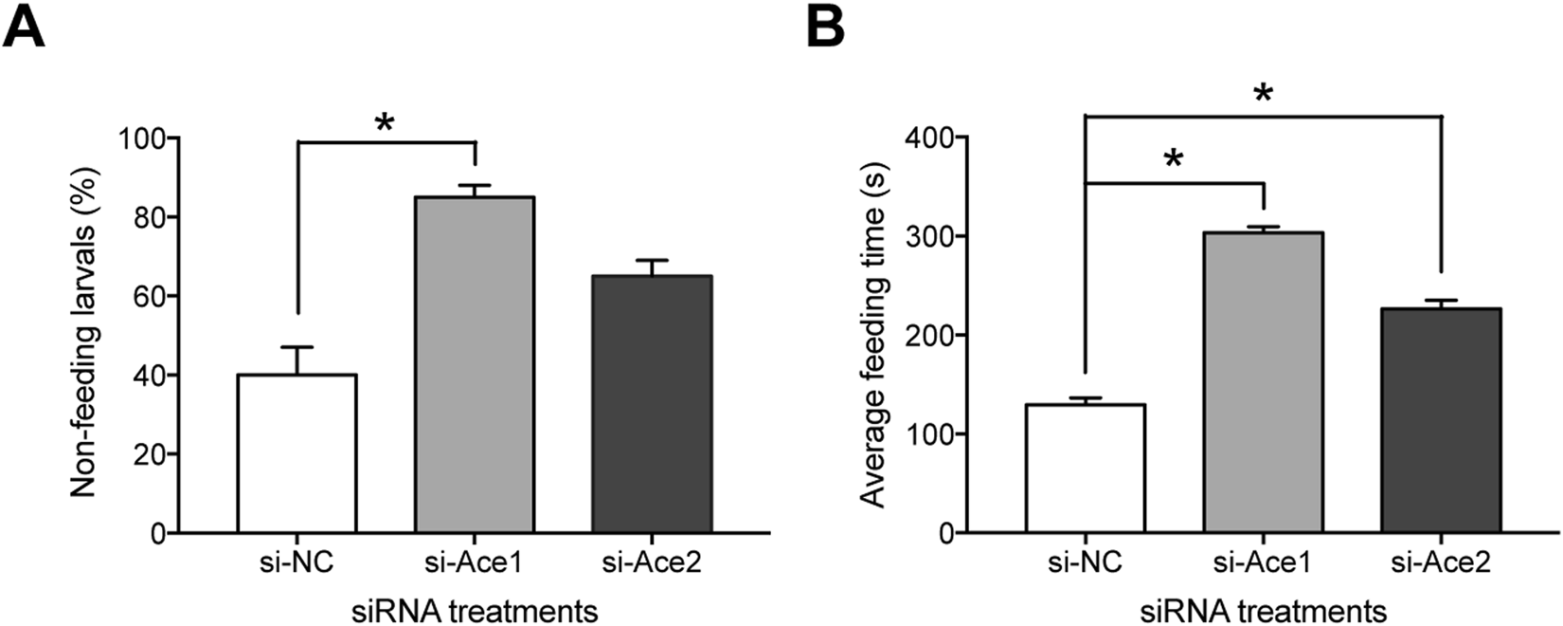
siRNA treatments led to reduced motor activity at 120 h PI. Panel A: Percent of larvae not able to move to the mulberry leaves after 20 mins. The histogram bars indicate percent of treated larvae that were unable to move to the leaves. The error bars indicate 1 SEM. * Significant difference at P ≤ 0.05. Panel B: The mean time (s) required to move to the mulberry leaves. The histogram bars indicate mean times and the error bars indicate 1 SEM. The asterisks indicate significant differences. All experiments were repeated in triplicate, n = 10.

We conducted a movement test to investigate the motor ability of survivors in the experimental groups. At 120 h PI, ten silkworms from each treatment group were starved for 24 h and then offered fresh mulberry leaves placed 1.5 cm away from them (Supplementary Fig. 2). After 20 mins, 40% of the controls larvae, 85% of the si*BmAce1*-treated larvae and 65% of the si*BmAce2*-treated individuals were unable to move to the mulberry leaves (Fig. 4A). For the larvae that reached the leaves, the mean time required to consume them was 129 s in the control group, 303 s in the si-*BmAce1* treated group and 226 s in the si-*BmAce2* group (Fig. 4B).

### *Ace* knockdown led to arrested larval and pupal development

**Figure 5:**
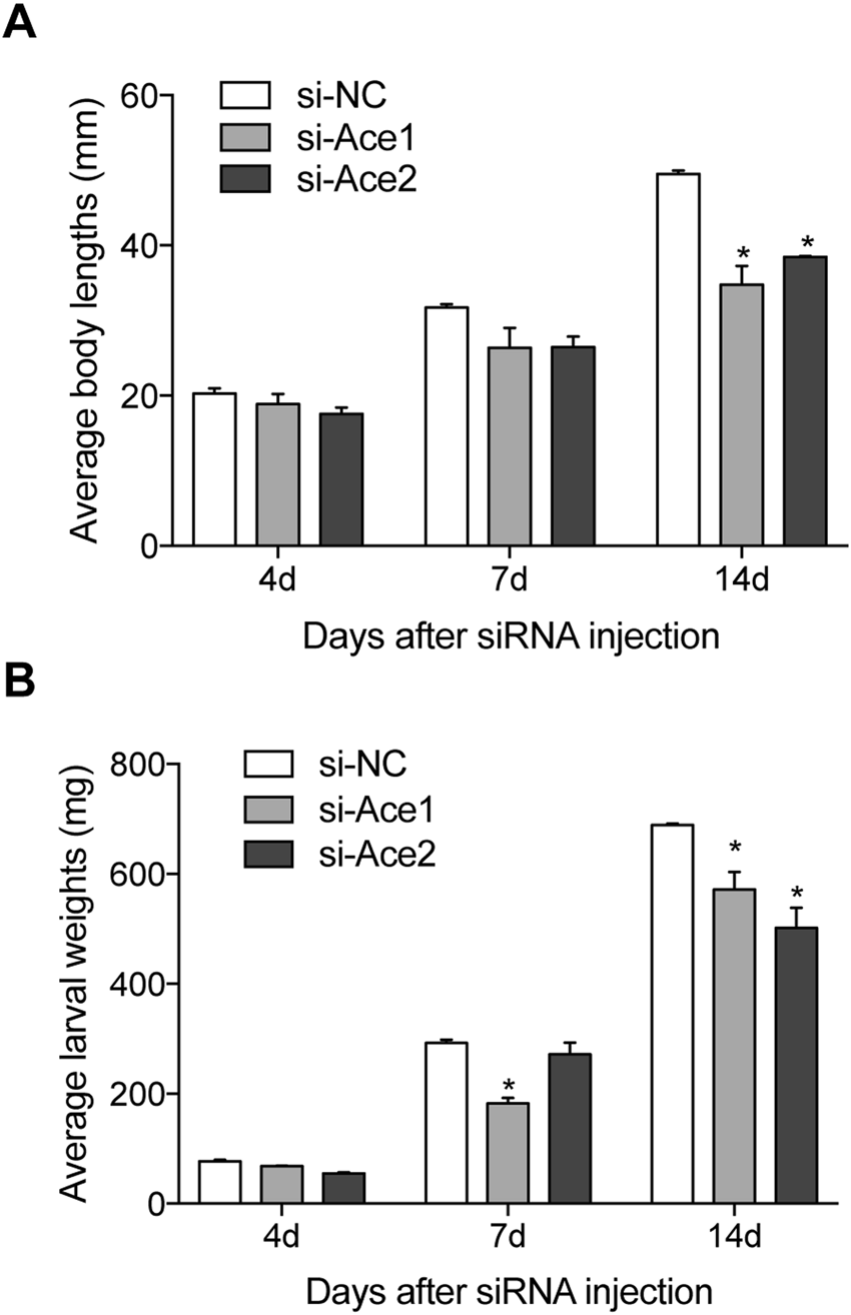
The siRNA treatments influenced larval development. Panel A: Mean larval body lengths of siRNA-treated silkworms at the indicated d PI. The histogram bars indicate mean body lengths and the error bars indicate 1SEM. * Significant difference at P ≤ 0.05. (B) Mean larval weight of siRNA treated silkworms at 4, 7 and 14 d PI. The histogram bars indicate mean body wet weights and the error bars indicate 1SEM. * Significant difference at P ≤ 0.05. All experiments were repeated in triplicate, n = 30.

**Figure 6:**
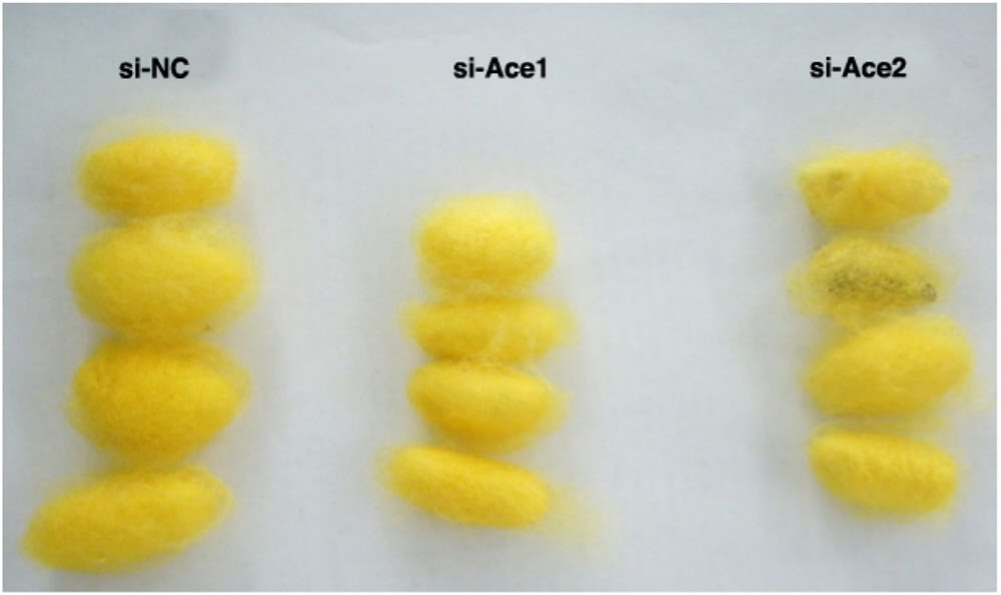
Cocoons of silkworms at 21 d after the indicated treatments.

We recorded body lengths and weights of larvae at 4, 7, and 14 d PI. These parameters did not change at 4 and 7 d PI, and were decreased relative to controls at 14 d PI. Mean body lengths were about 35 mm (si*BmAce1-*treated group) and 38 mm (si*BmAce2*-treated group), significantly shorter compared to the control group (49 mm; Fig. 5A). Similarly, mean larvae weights were reduced in both experimental groups (about 571 mg in the si*BmAce1* group and 505 mg in the si*BmAce2* treated group at 14 days PI, lower than the control group (689 mg; Fig. 5B). The siRNA treatments led to smaller cocoons in the si*BmAce1*-treated group and to silk-lacking cocoons in the si*BmAce2-*treated group (Fig. 6).

## Discussion

AChE has been widely used as a pharmaceutical and insecticide target. It is the target of therapeutic agents against helminth parasites^35^. Tacrine, a cholinesterase inhibitor, used for the treatment of Alzheimer’s disease symptoms^36^. Many insecticides and acaricides target AChE to minimize crop loss. A large number of these compounds have been withdrawn because of their high environmental damage, including loss of non-target organisms and high pest insect resistance. Investigating insect *ace* genes and the proteins they encode is necessary to develop pesticides that are more target-selective and compatible with resistance management programs.

There are four genes encoding AChE in the nematodes *Caenorhabditis elegans* and *C. briggsae* ^37^. Three of them encode distinct enzymes whereas the fourth is likely to be non-catalytic^35^. AChE-3 acts in resistance to phoxim, an OP used in veterinary medicine and the other AChEs presumably operate in other areas of *C. elegans* physiology^38^. The nematode *ace*s have different expression patterns in muscle, sensory neurons and motor neurons, from which we infer they have non-overlapping functions. The two insect *ace*s also have different expression patterns and physiological functions^9, 12, 17, 21, 34^. Knockdown of either gene led to high death rates in experimental insects, indicating both genes have essential, and separate, actions. In rice stem borer, *C. suppressalis*, *CsAce1* encodes an AChE that acts in sensory, motor and larval growth physiology. Silencing *CsAce1* resulted in high mortality, motor disability and retarded development. *CsAce2* may make specific contributions to sensory physiology because suppressing it led to mortality without influencing larval growth or motor ability^12^. However, in the diamondback moth, *P. xylostella*, *PxAce2* acts in motor ability and larval growth although mRNA encoding it is less abundant than mRNA encoding *PxAce1*. Knockdown of either gene led to death, motor disability and slowed larval development^34^, indicating the biological significance of these enzymes.

*B. mori* has been domesticated for more than two thousand years. Here, we infer that domestication influenced the biological assignments of the *ace*s. Based on expression patterns and RNAi results, the *BmAce1* transcript was abundant specifically in muscle, where it acts in motor control and larval growth. *BmAce2* was expressed in all tissues we analyzed and most likely acts in sensory physiology. This is consistent with our reports that *ace2* encodes the major AChE in silkworms, whereas *ace1* encodes the major AChE in other non-domesticated lepidopterans, such as *C. suppressalis* and *P. xylostella*. We hold the view that these differences between *B. mori* and other lepidopterans emerged after the silkworms were domesticated.

We found that the average body lengths of larvae treated with si*BmAce1* and si*BmAce2* were similar at 7 days PI, but, in terms of weight accumulation, si*BmAce1*-treated larvae were lighter at 7 d PI. Si*BmAce2*-treated larvae were longer, but lighter than si*BmAce1*-treated group. These results indicate to us that the body length, based on the photographic results, did not positively correlate with weights. These results further support our conclusion that knockdown of *ace* genes disrupts metabolic processes broadly. Overall, the *ace1* orthologs have similar functions over a broad range of insect taxa and *ace2* have generally evolved different biological functions. We found *ace* knockdown led to thinner and smaller cocoons, we take this finding to indicate the AChEs also act in silk production.

The RNAi efficiencies of two *ace* genes at the transcriptional level differed and knockdown of *BmAce2* seemed to be more effective. However, we found the AChE activities of si*BmAce1-* and si*BmAce2*-treated insects were similar. We speculate the differences between siRNA efficiencies could be balanced by differences in protein stability over time and/or differences in AChE protein catabolism. Looking more broadly, we expect that understanding the molecular mechanisms of how these genes influence cocoon production will lead to new hypotheses and research results of value in the silk production industry.

## Methods

### Insects

The silkworms used in the experiments were breed 7091, kindly provided by Sericultural Research Institute (Zhenjiang, Jiangsu), Chinese Academy of Agricultural Sciences. First and 2^nd^ instar larvae were maintained at 28 °C, remaining stages at 24–26 °C under 16 L:8D photoperiod and 60–80% relative humidity.

### siRNA design and injection

si*BmAce1* and si*BmAce2* were designed for gene-specific silencing experiments. To avoid off-target effects, the siRNAs were aligned with other *ace*s and were used to interrogate the silkworm genome by BLAST (http://www.ncbi.nlm.nih.gov/BLAST). The siRNAs that contained more than 11 contiguous base pairs of homology to the other *ace* or 16–17 contiguous base pairs of homology to other AChE protein coding sequences were discarded. siRNAs with shuffled si*BmAce1* and si*BmAce2* sequences were used as the negative control (Table 1).

**Table 1:** The sequences of siRNA for RNA interference and primers for quantitative real-time PCR.

	Name	Sequences
siRNA	Bm-*Ace*1-667	5′-GCAGAUAUAAUGGCAUCUATT-3′
		5′-UAGAUGCCAUUAUAUCUGCTT-3′
	Bm-*Ace*2-1526	5′-GCCCATCGTTATGCAGAAATT-3′
		5′-UUUCUGCAUAACGAUGGGCTT-3′
	Bm-*Ace*2-338	5′-GGGAAGGTGAGAGGAATTATT-3′
		5′-UAAUUCCUCUCACCUUCCCTT-3′
	Negative control	5′-UUCUCCGAACGUGUCACGUTT-3′
		5′-ACGUGACACGUUCGGAGAATT-3′
qPCR primers	Bm-RP49-F	5′-TGAACCCCCATACAGCGAATCC-3′
	Bm-RP49-R	5′-TCTCCGTGCCAACCAGAAATAGG-3′
	Bm-AChE1-F	5′-GAGGAAAGCATTTTACGAGGCACA-3′
	Bm-AChE1-R	5′-GTTCGTCAGCACTCTTCTTCCTTA-3′
	Bm-AChE2-F	5′-CATGTCCCTTCAGTACCATTCCC-3′
	Bm-AChE2-R	5′-CGCCGTGTATGTGTAGTAATGAGG-3′

siRNAs were chemically synthesized by Shanghai GenePharma Co., Ltd (Shanghai, China). To monitor the spread of siRNA in larval bodies under fluorescence microscopy, both constructs were fluorescently labelled with fluorescein amidite at the 5′end of the sense strand. A final siRNA concentration (1 μg/μl) was obtained by dissolving them in RNase-free water (Milli-Q grade). We used an Eppendorf InjectMan NI 2 microinjection system (Eppendorf, Hamburg, Germany) to inject 1 μl siRNA between the third and fourth segments of 1 d, 3^rd^ instar larvae. The injection pressure was 200 Pa and injection time was 0.2 s. The needles were made by a micropipette puller (Model P-87, Sutter Instruments Co., Novato, CA, USA) with parameters: Heat = 596, Pull = 0, Vel = 30–40, Time = 250 s. To avoid construct leakage, needles were kept still at the injection point for 30 s. All experiments were repeated in triplicate, n = 30 larvae/treatment.

### Total RNA isolation and cDNA synthesis

The tested larvae were frozen in liquid nitrogen and stored at −80 °C for further use. Total RNA was isolated with TRIzol reagent (Takara, Kyoto, Japan) following the recommended procedures. Genomic DNA was removed by treating total RNAs with DNAse I following the manufacturer’s protocol (Ambion, Austin, TX, USA). The RNA quality was checked on a 1.5% agarose gel. The first strand of the cDNA template was synthesized with Maloney murine leukemia virus reverse transcriptase (Promega, Madison, WI, USA), using Oligo (dT_18_) as the anchor primer and 1 mg of total RNA as the template.

### qPCR

The qPCR reactions were carried out using an ABI Prism 7000 with SYBR Premix Ex Taq^TM^ (Takara). The qPCR primers (Table 1) were designed using PrimerQuest (http://www.idtdna.com/Scitools/Applications/Primerquest/). The stably expressed Ribosomal Protein 49 (*RP49*) was used as the reference gene^39^. The qPCR program included one cycle of 95 °C for 10 s, 40 cycles of 95 °C for 5 s and then annealed at 60 °C for 31 s, followed by one cycle of 95 °C for 15 s, 60 °C for 60 s, 95 °C for 15 s and 60 °C for 15 s. The qPCR specificity was monitored with melting curve analysis and gel electrophoresis. Amplification efficiencies were determined by a series of template dilutions. The relative abundances of mRNAs was calculated using the 2^−△△CT^ method^40^.

### AChE activity

Enzyme activities were measured using the kinetic method with minor modifications^20^. Two silkworms were homogenized on ice in 1 ml phosphate buffer (0.02 M, pH 7.5, 0.1% Triton X-100) in a glass tissue grinder. The homogenate was centrifuged at 10,000 g for 20 m at 4 °C and the supernatants were used as the enzyme source. 50 μl enzyme preparation was added to well of a microplate, along with 50 μl phosphate buffer (pH 7.5, 0.02 M) and 50 μl Ellman’s reagent (5,5′-dithiobis-(2-nitrobenzoic acid; 45 μM). The reactions were started by adding 50 μl acetylthiocholine iodide (1.56 mM) at 25 °C. The reactions were monitored using a Bio-Rad microplate reader at 405 nm every 30 s for 20 min. The reaction linearity was > 0.9. Protein concentration was measured with the Bradford method using bovine serum albumin as the quantitative standard^41^.

### Motor experiment

At five d PI silkworms were fasted for 24 h, then fresh mulberry leaves were placed 1.5 cm away from 10 silkworms for each experiment. The time required to move to the mulberry leaves and numbers of silkworms that did not reach the leaves within 20 mins were recorded. All experiments were repeated in triplicate.

### Larval growth and death rates

Silkworm body lengths and larval weights were recorded at 4, 7, and 14 d PI. At 21 d PI individuals that did not move when touched with a Chinese brush were recorded as dead. The mortalities of siRNA-treated silkworms were recorded at 24 h, 48 h, 72 h, and 96 h PI.

### Statistics

All experiments were repeated in three biologically independent replicates. The data were analyzed with Student’s t-test using SPSS v22.0 software (SPSS, Chicago, IL, USA). Differences were considered statistically significant at p < 0.05.
